# Mesmerism

**Published:** 1846-08

**Authors:** 


*Mesmerism.*—From a letter published in a Dublin paper, it appears that the £100 note deposited for six months in the bank of Messrs. Ball and Co., which was, according to the terms of the advertisement in the public papers, "to become the property of any person who, without opening the envelope in which it was contained, should describe every particular respecting the note—such as its number, its date, the bank at which it was payable, &c., and who should read three English words, plainly written on a slip of paper, which was contained in the same envelope with the note," has not been awarded. The six months expired on the 31st March, but the time was extended to the 18th of April, to meet the convenience of a lady, a professor of mesmerism, and the authoress of an ingenious book on the subject, who arrived from London in the beginning of the month, and who expressed a wish to have some time longer to prepare her *clairvoyance* for the test. Six months and seventeen days having expired, and no person having appeared at the bank to examine the envelope, it was opened on the 18th instant, in the presence of Messrs. Ball and Doyne, and one or two other persons connected with the establishment. The note proved to be a printed cheque issued by the house of Messrs. Ball and Co. for £100, payable to Œdipus or bearer, and dated the 1st of October, 1845. The English words (written on a separate slip of paper) were, "To Œdipus alone." Although no person applied at the bank to inspect the envelope containing the note, some communications were received from different parts of England, and one from America, (but none from Ireland,) containing mesmeric revelations respecting the number of the note; and one letter (from Plymouth,) enclosed a picture, or (intended) *fac simile* of it. It is unnecessary to add, that these mesmerically-inspired persons were mistaken in every particular.—*Prov. Med. and Surg. Journ.*

